# Non-Neuronal Cells in the Hypothalamic Adaptation to Metabolic Signals

**DOI:** 10.3389/fendo.2017.00051

**Published:** 2017-03-21

**Authors:** Alejandra Freire-Regatillo, Pilar Argente-Arizón, Jesús Argente, Luis Miguel García-Segura, Julie A. Chowen

**Affiliations:** ^1^Department of Endocrinology, Hospital Infantil Universitario Niño Jesús, Instituto de Investigación la Princesa, Madrid, Spain; ^2^Department of Pediatrics, Facultad de Medicina, Universidad Autónoma de Madrid, Madrid, Spain; ^3^Centro de Investigación Biomédica en Red: Fisiopatología de la Obesidad y Nutrición (CIBEROBN), Madrid, Spain; ^4^IMDEA Food Institute, Campus of International Excellence (CEI) UAM + CSIC, Madrid, Spain; ^5^Laboratory of Neuroactive Steroids, Department of Functional and Systems Neurobiology, Instituto Cajal, CSIC (Consejo Superior de Investigaciones Científicas), Madrid, Spain; ^6^Centro de Investigación Biomédica en Red de Fragilidad y Envejecimiento Saludable (CIBERFES), Madrid, Spain

**Keywords:** hypothalamus, metabolism, energy, homeostasis, glia, inflammation

## Abstract

Although the brain is composed of numerous cell types, neurons have received the vast majority of attention in the attempt to understand how this organ functions. Neurons are indeed fundamental but, in order for them to function correctly, they rely on the surrounding “non-neuronal” cells. These different cell types, which include glia, epithelial cells, pericytes, and endothelia, supply essential substances to neurons, in addition to protecting them from dangerous substances and situations. Moreover, it is now clear that non-neuronal cells can also actively participate in determining neuronal signaling outcomes. Due to the increasing problem of obesity in industrialized countries, investigation of the central control of energy balance has greatly increased in attempts to identify new therapeutic targets. This has led to interesting advances in our understanding of how appetite and systemic metabolism are modulated by non-neuronal cells. For example, not only are nutrients and hormones transported into the brain by non-neuronal cells, but these cells can also metabolize these metabolic factors, thus modifying the signals reaching the neurons. The hypothalamus is the main integrating center of incoming metabolic and hormonal signals and interprets this information in order to control appetite and systemic metabolism. Hence, the factors transported and released from surrounding non-neuronal cells will undoubtedly influence metabolic homeostasis. This review focuses on what is known to date regarding the involvement of different cell types in the transport and metabolism of nutrients and hormones in the hypothalamus. The possible involvement of non-neuronal cells, in particular glial cells, in physiopathological outcomes of poor dietary habits and excess weight gain are also discussed.

## Introduction

Our understanding of the neuronal circuits controlling metabolism has advanced in recent years and progress has been made in the development of potential treatments for obesity, particularly in specific monogenic forms of obesity (1). However, the brain is not composed of neurons alone; other cell types actually outnumber these electrically excitable nerve cells and participate in and/or modulate all neuronal functions. In the hypothalamus, this includes the participation of non-neuronal cells in the modulation of neuronal circuits controlling appetite and metabolism.

Non-neuronal cells in the central nervous system (CNS), including glia, epithelial cells, pericytes, and endothelia, perform a wide spectrum of functions throughout the brain. Many of these functions are common in each brain area, although the specific outcomes are at least in part dependent on the neuronal circuits that are affected by their actions. Moreover, within each class of non-neuronal cell type there are generalized subclassifications that, although quite incomplete, indicate diverse functional states. There are also specialized cell types found only in specific areas of the brain. One important example that will be discussed in greater detail is tanycytes, specialized glial cells found lining the third ventricle and in close proximity to the neuroendocrine hypothalamus. The fact that there is wide heterogeneity within each non-neuronal cell type has become increasingly clear; however, we currently do not have the tools available to sufficiently distinguish between these subpopulations and this has clearly hindered advances in this field.

With the explosion in the prevalence of obesity that has occurred almost worldwide, investigation in the area of metabolic control has become a priority. This has led to an increase in our understanding of how non-neuronal cell types participate in the neuroendocrine control of appetite and energy expenditure, as well as in the response to increased weight gain and the development of secondary complications. Here, we have briefly outlined the different types of non-neuronal brain cells and some of their functions, both in general and those that are specific to the hypothalamus and metabolic circuits.

## Classification of Non-Neuronal Cells in the Brain

### Astroglial Cells

**Figure 1:**
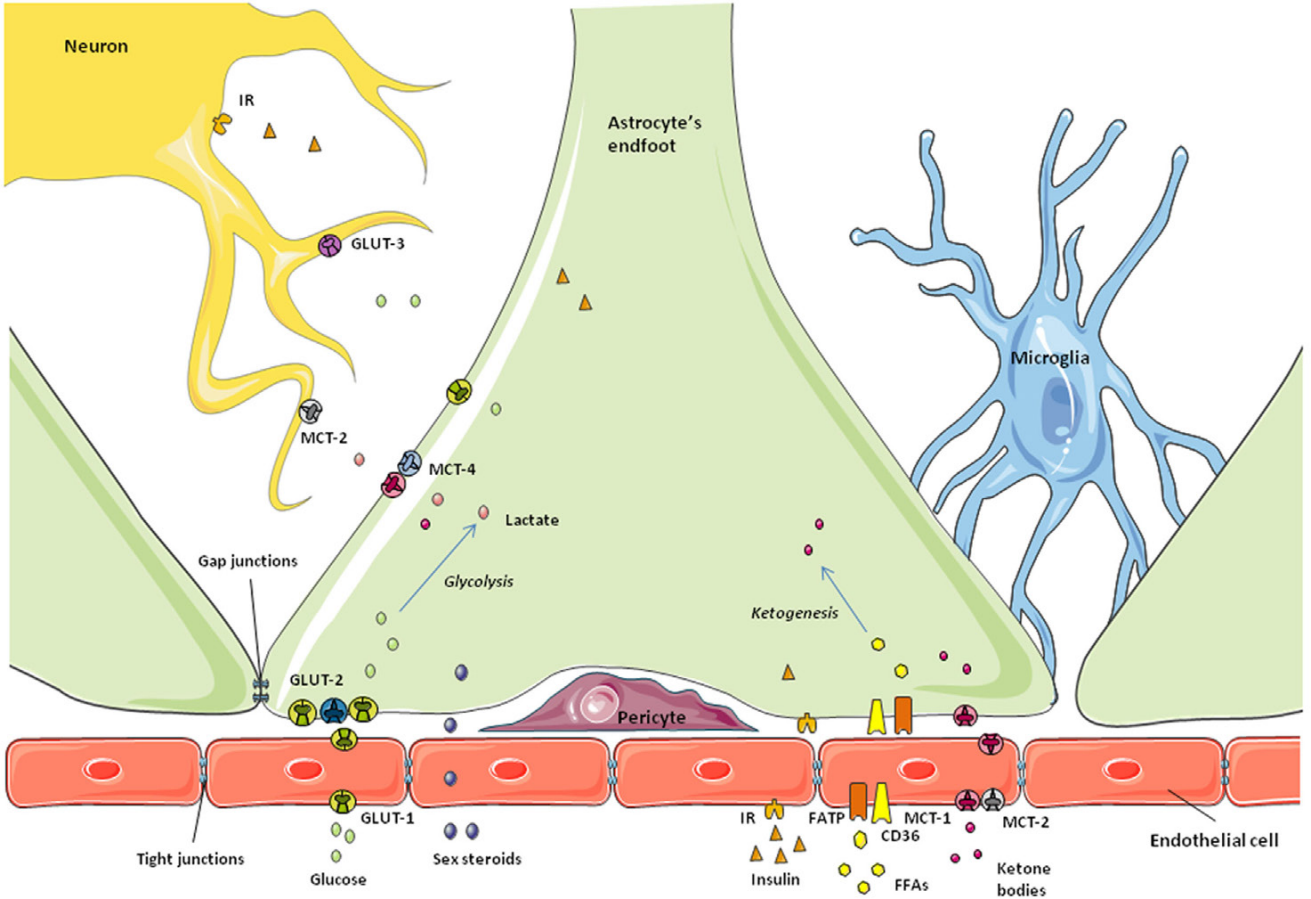
**Schematic representation of the blood–brain barrier**. Astrocytic endfeet surround the microvessels and take up the nutrients and metabolic factors coming from the bloodstream. Endothelial cells at this level express specific receptors and transporters and restrict the passage of small molecules to the brain due to the tight junctions between them. Depending on the metabolic state, nutrients and factors are processed by astrocytes to control their access to neurons and maintain brain homeostasis.

Astrocytes were the first class of glial cells to be described (2) and they are also the most studied. One example of this is that a search of the word “astrocyte” in the PubMed Central database obtains approximately 48,000 results; typing “microglia” or “oligodendrocyte” receives less than 30,000 returns in either case. Astroglia are also the most abundant cell type in the CNS and were first thought to only constitute the physical and metabolic support for neuronal function (2). We now know that they are much more than just “neuron helpers” (3). Astrocytes do indeed transport nutrients and metabolic factors across the blood–brain barrier (BBB) and release them to the extracellular fluid where they can be used by neurons and other glial cells (4, 5) (Figure 1). However, it is now clear that this supply of energy substrates to other cell types is regulated with astrocytes responding to metabolic changes in order to maintain brain homeostasis (6–9). Astrocytes are also the only glial cells known to store energy through glycogenesis (10). In the synaptic cleft, they reuptake neurotransmitters and also can release gliotransmitters, forming part of what is called the “tripartite synapse” (11, 12). At the level of the BBB, astrocytes are involved in the formation and maintenance of some of the barrier properties (13) and can regulate vasodilatation, thus controlling the flow of blood-borne substances (14, 15).

Astrocytes are connected by gap junctions in their plasma membranes, which enable direct transport of small molecules between cells. Initially, it was thought that these channels allowed passive diffusion of substances; however, the transport through gap junctions is tightly regulated (16, 17). One important function of these gap junctions is the rapid transmission of calcium waves within the glial network, resulting in a form of non-neuronal signal transmission (18).

When employing classical labeling methods, astrocytes appear to have a star-shaped morphology, although two different forms, protoplasmic and fibrous, can be distinguished. The first are mainly found close to synapses and blood vessels, whereas the latter are frequently found within the white matter (19–21). The morphology of these glial cells also changes in respect to their functional or activational state. The fact that astrocytes differentially express certain proteins (e.g., receptors, enzymes, channels, etc.) depending on the brain area and the physiological or pathophysiological conditions to which they are subjected raises questions regarding the current definition and classification of astroglial cells (22). Growing evidence indicates that astrocytes are vastly heterogeneous (23–28). For example, Matthias and colleagues reported that within the hippocampus subsets of GFAP expressing cells expressed either glutamate transporters or glutamate receptors (23). Moreover, astrocytes throughout the brain differentially express connexins (24) and GABA and glutamate receptors (26) and different astrocyte populations are reported to differentially support developmental functions and synapse formation (28, 29). Thus, our understanding of the functions of astrocytes is advancing, but much is yet to be learned. Indeed, we are only now beginning to have the tools to understand the grand diversity of these glial cells.

### Microglia

Microglial cells constitute the bulk of the immune system in the brain. There have been different systems suggested for the classification of microglia, with most engaging morphological features. The most general classification includes an amoeboid form, characteristic of early development, and a ramified form or “resting” microglia and reactive microglia (30–32). The phenotype of reactive microglia is defined by changes in morphology, to short and thick projections, and the release of factors like cytokines, nitric oxide, and reactive oxygen species (30, 31, 33, 34). This “activation” or change in phenotype can occur in response to brain damage, toxic substances, or injury due to harmful conditions like obesity or a high fat diet (HFD) (35, 36) and when this state is sustained, it can lead to a pathological chronic state of reactive microgliosis (37). However, the division that separates resting and reactive microglia has become more diffuse as we learn more about these cells (38).

One of the main functions of microglia is to “clean” the CNS by phagocytosis of cellular debris, foreign matter, and other wastes (39). In this manner, they participate in development and synaptic plasticity (40–42). They can also release gliotransmitters and metabolic factors, contributing to maintain brain homeostasis (38, 43). Importantly, as part of the immune system, microglial cells respond to injury and harmful factors, including fatty acids, by releasing cytokines and to infection by presenting antigens to T-cells (35, 39, 43).

### Oligodendrocytes

Oligodendrocyte projections wrap neuronal axons, forming the myelin sheaths in the CNS. To date, no direct link between these cells and systemic metabolic function has been verified, although some studies connecting metabolic signals with changes in myelination or oligodendrocyte survival suggest at least an indirect relationship with metabolism (44–48). However, it has been recently shown that oligodendrocyte precursors (NG2 glia) in the median eminence are important for the function of leptin receptor-expressing neurons, whose dendritic processes they contact (49).

### Tanycytes

**Figure 2:**
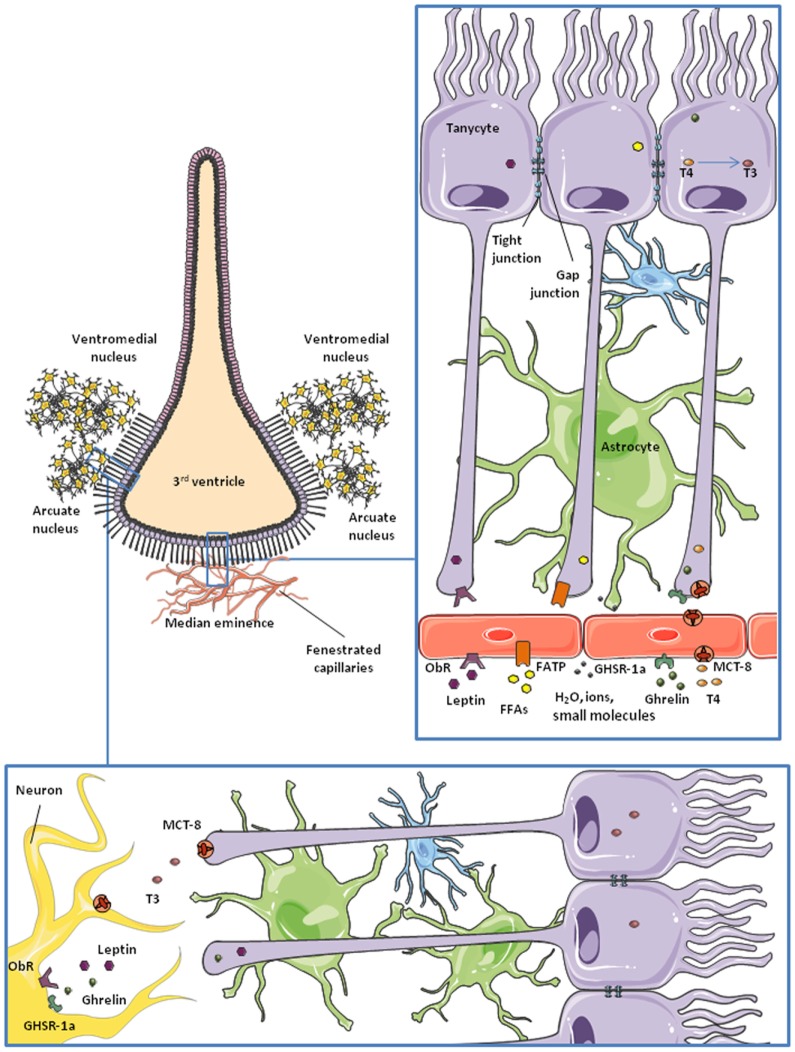
**Schematic representation of tanycytes lining the third ventricle**. Microvessels of the median eminence are fenestrated, so they allow water, ions, and small molecules to freely enter the brain. β2-tanycytes present tight junctions between them, forming a blood–CSF barrier. They take up nutrients and factors from the microvessels and control their access to the cerebrospinal fluid (CSF) and the rest of the brain. This is probably the main route for some hormones and nutrients to the hypothalamus. Also, tanycytes are communicated through gap junctions, so some molecules can be transported to lateral β1 and α-tanycytes and gain access to the arcuate nucleus and the ventromedial nucleus of the hypothalamus.

These specialized ependymal-like glial cells lining the ventral and ventrolateral part of the third ventricle (Figure 2) are proving to be very interesting as we know more about them. From dorsal to ventral, they are classified as subtypes α1, α2, β1, and β2. They are polarized cells: on the ventricle-side they express numerous receptors and transporters in their membrane and can be ciliated (not β2 tanycytes); and on the opposite side they present a long process that projects into the hypothalamic parenchyma or the median eminence (50). The β2 tanycytes can be found close to the median eminence, a subhypothalamic circumventricular organ. Capillaries on the median eminence are fenestrated, making the BBB permeable to many substances (50–52). The long processes of β2-tanycytes project into these fenestrated vessels, forming a blood–CSF barrier (BCSFB). The tight junctions between them, in addition to the specific transporters that they express, allow them to control the entry of many substances into the hypothalamus (53). They can also regulate the permeability of this barrier at this level of the brain, by the release of vascular endothelial growth factor-A in response to metabolic changes (54) and possibly by other mechanisms (55). Although astrocytes are the major cells expressing gap junctions, tanycytes also express these structures and can also produce calcium wave signaling (56). Tanycytes also possess stem cell properties (57) and participate in glutamate recycling (58), nutrient sensing (59, 60), and the conversion of thyroid hormones (TH) (61).

### Pericytes

Pericytes are contractile cells surrounding the blood vessels (62, 63). In addition to their ability to modify blood flow due to their contractibility, brain pericytes have multiple roles in the development and maintenance of the BBB (64, 65), including macrophage-like functions and characteristics (66–68), angiogenic properties (69), and a role in neuroinflammation (70). Indeed, in response to brain injury, there is evidence that pericytes change to a microglia-like phenotype (68, 71), migrate to the brain parenchyma (72), and are involved in scar formation (73), antigen presentation (74), and the release of inflammatory factors (75, 76). Pericytes are also reported to be multipotential stem cells in the CNS (77). However, the identity of these stem cells is still a subject of controversy (78), due to the lack of reliable pericyte markers (79).

### Endothelial Cells

Endothelial cells, along with pericytes, form the walls of the microvessels, taking part in the transport of metabolites through the BBB (80). The particularities of BBB endothelial cells, described below, allow for a strict control of the passage of substances from the blood into the CNS.

### Epithelial Cells/Ependymocytes

In the CNS, epithelial cells can be found in the choroid plexus and lining the ventricles. They secrete the cerebrospinal fluid (CSF) that fills the ventricles and factors involved in neurogenesis and brain development (81–84). Epithelial cells of the CNS also express transporters for glucose, amino acids, and other molecules (85–87), as well as receptors for hormones such as sex steroids (88–90) and leptin (91). Moreover, they form a type of BCSFB due to the tight junctions between them (92). Ependymal cells are epithelial cells lining the ventricles. Their polarized organization and beating of numerous cilia are important for the movement of CSF (93, 94). They also possess precursor properties and, together with tanycytes, form the hypothalamic neurogenic niche (95).

## Functions of Non-Neuronal Cells

### Transport of Metabolic Signals into and within the Hypothalamus

The transport of nutrients and other metabolic signals is one of the best studied functions of non-neuronal cells in the nervous system. At the physiological level, nutrients from the diet, hormones, and other substances are delivered to all tissues through the bloodstream. However, due to its exceptional importance and vulnerability, the CNS protects its homeostasis by carefully controlling what can and cannot enter from the circulation. This function is carried out by the BBB, which is formed by specialized glia, pericytes, and endothelial cells expressing transporters, receptors, and sensors that allow them to select the information and nutrients accessing the nervous tissue (80) (Figure 1). As nutrients and metabolic signals are also found in the CSF, there is a BCSFB, formed by ependymal cells and tanycytes, in the third ventricle (50, 96, 97) (Figure 2). The distribution of tight junction proteins between tanycytes at this level is important in determining the permeability of the barrier, being lower at the median eminence, where there are fenestrated capillaries and higher next to the arcuate nucleus (98).

The first checkpoint for any substance to cross the BBB into the CNS is the endothelial cell, the bricks forming the capillary walls (Figure 1). Endothelial cells in the BBB are phenotypically different from those of peripheral vessels and restrict the access of blood-borne substances to the extracellular fluid of the CNS (80, 99). To achieve this, these endothelial cells have tight junctions between them, reduced endocytosis, no fenestrations, and specific transporters and receptors, in addition to a large number of mitochondria (65). Thus, brain capillary endothelial cells broadly determine the barrier permeability. Surrounding these capillaries are the astrocytic endfeet, along with pericytes and microglia (Figure 1). These other cells also participate in the regulation of nutrient and hormone entry, and thus metabolic signaling, from the periphery (80, 99, 100). Astrocytes and other non-neuronal cells can detect changes in the concentrations of specific nutrients and the presence of other signals and react consequently to maintain brain homeostasis, as described below.

#### Glucose

Glucose, the main energy source of the CNS, enters the brain from the bloodstream crossing the BBB through specific transporters. As normal brain function depends on its glucose supply, this step is highly regulated. That is, the transport of glucose across the BBB adapts in response to cerebral energy demand in order to maintain glucose homeostasis in the brain. The facilitative glucose transporter (GLUT)-1 is largely responsible for glucose transport across the BBB. This protein is expressed in non-neuronal cells throughout the CNS, especially in astrocytes and endothelial cells of the BBB (101), as well as in tanycytes along the BCSFB (50). However, GLUT-1 in endothelial cells is highly glycosylated, having a higher molecular weight than the isoform expressed in astrocytes and other glial cells (101, 102). As indicated in a recent review, some authors suggest different functional characteristics between the two forms of GLUT-1, although there is no consensus on this subject (103).

Changes in glucose concentration are rapidly detected in the hypothalamus, which adapts to such variations and emits a response to maintain glucose homeostasis not only in the brain, but also systemically as glucose-sensing neurons in the hypothalamus send signals to the autonomous nervous system, reaching peripheral organs such as the pancreas or the liver (104–107). There is more than one mechanism for central glucose sensing and different cell types are involved in this essential task (107–110). Two populations of glucose-sensing neurons have been identified: glucose-excited and glucose-inhibited neurons (GE and GI, respectively) (111) and glial cells also participate in these important glucose-sensing mechanisms. Astrocyte endfeet express GLUT-2 which, in addition to its transport functions, participates in glucose sensing (110, 112). This GLUT is highly expressed in tanycytes along the BCSFB (109), with these specialized glial cells also participating in glucose-sensing processes. In addition to expressing GLUT-2, astrocytes and tanycytes express sodium glucose transporter (SGLT)-1, glucokinase (GCK), and K_ATP_ channels (110), proteins that are all known to be involved in glucose-sensing mechanisms. Indeed, the classical mechanism for glucose sensing in pancreatic β-cells requires glucose uptake through GLUT-2 in rodents or GLUT-1 in humans, GCK, and activation of ATP-sensitive K^+^ channels (112, 113). This system shares some similarities with glucose-sensing pathways in astrocytes and tanycytes.

One proposed model for glucose sensing in tanycytes involves glucose entering the cell through GLUT-2 and phosphorylation by GCK. Subsequently, glucose-6-phosphate undergoes glycolysis, producing pyruvate and, through the action of lactate dehydrogenase, lactate. Lactate is transported to the extracellular space by monocarboxylate transporter (MCT)-4 or MCT-1, and then taken up by neurons through MCT-2 (109). Depending on the kind of neuron, GE or GI, an excitatory or inhibitory signal will be produced in the hypothalamus and sent to other brain areas and the autonomic nervous system (108). Tanycytes can also respond rapidly to glucose and other inputs by producing calcium waves, a process requiring ATP release and autocrine signaling through purinergic P2Y receptors (56, 59). The precise mechanisms involved in this tanycytic response are not yet fully elucidated, but it constitutes a possible model for tanycyte–neuron interaction.

Glucose sensing in astrocytes involves a similar process. Indeed, according to the “astrocyte-neuron lactate shuttle” hypothesis proposed by Pellerin and Magistretti over two decades ago (4), lactate from glucose or glycogen metabolism released by astrocytes is not only used by neurons as an energy source but can also signal energy availability to glucose-sensing neurons. Glucose transport into astrocytes is facilitated by GLUT-2 or occurs through gap junctions in a passive manner (112, 114–116). This glucose can be metabolized or stored as glycogen. However, it is still debated as to whether astrocytes secrete only lactate or also glucose to the extracellular fluid to act on glucose-sensing neurons and to be used as fuel (112). Moreover, astrocytes and tanycytes can respond to an increase in glucose or to other signals (i.e., some neurotransmitters) by secreting endozepines, anorexigenic peptides that act on hypothalamic neurons to maintain energy homeostasis (107, 117) and that also participate in unsaturated long-chain fatty acid metabolism in astrocytes (118).

The precise mechanisms of glucose transport and sensing in the hypothalamus are yet to be fully elucidated. For example, SGLT, an active sodium co-transporter, is reported to be involved in glucose sensing in the ventromedial nucleus of the rodent hypothalamus (119), although it is not clear whether this sensing occurs in glucose responsive neurons or in astrocytes. By using genetically engineered mouse models, García-Cáceres and collaborators recently demonstrated that insulin signaling in astrocytes plays a role in the regulation of systemic glucose homeostasis. Specific ablation of the insulin receptor (IR) in astrocytes was shown to impair their uptake of glucose and the ability to correctly respond to changes in glycemia (120). Other studies suggest a role of leptin in increasing (121) or ghrelin in reducing (122) glucose uptake by astrocytes, which might also affect glucose sensing. It thus appears that the transport of glucose by astrocytes is highly regulated by diverse nutrient and hormonal signals.

#### Ketone Bodies

Monocarboxylates are molecules with one carboxylate group; some examples with metabolic functions include not only lactate, but also pyruvate and ketones, all of which can be used by neurons as an alternative energy source in addition to acting as metabolic signals (123–126). The brain expresses MCTs-1, -2, and -4, with MCT-1 being found in endothelial and ependymal cells, as well as in astrocyte endfeet at the BBB (127, 128). MCT-2 is expressed in endothelial cells, but not in astrocytes, whereas MCT-4 appears to be specific for astrocytes (58, 129–131). Ketone bodies and other monocarboxylates from the bloodstream cross the BBB through specific MCTs present in both the luminal and abluminal sides of the endothelial cells (132, 133). Astrocyte endfeet not only takes up monocarboxylates through MCT-1 (132–134), but these glial cells are also able to synthesize ketone bodies from fatty acid β-oxidation and secrete them as an energy source for neurons and other glial cells (Figure 1). Tanycytes have also been suggested to transport lactate through MCT-2 in a photoperiodic model of Siberian hamster (58). These authors found that MCT-2 and the glutamate transporter GLAST were decreased during a short photoperiod, which could indicate a change in seasonal neurotransporter supply. In the rat brain, tanycytes were shown to express functional MCT-1 and MCT-4 in an anatomically specific manner (135), suggesting that these glial cells may also participate in lactate transport to neurons.

Regulation of the transport and production of ketone bodies in the brain is important in metabolic control as hypothalamic sensing of these monocarboxylates also participates in the regulation of food intake (126, 136). Indeed, after the initial HFD-induced hyperphagia, there is a reduction in food intake that is reported to be mediated, at least in part, by ketone body signaling to hypothalamic neurons. These ketone bodies are synthesized by hypothalamic astrocytes as products of fatty acid metabolism (136, 137).

#### Lipids

Lipid sensing in the hypothalamus is necessary for the correct regulation of energy balance (138). There are lipid sensing neurons that are excited or inhibited by fatty acids, depending on the type of neuron and also the metabolic state, i.e., fasting versus overfeeding (139). Although the role of glial cells in this process is not fully understood, astrocytes are the primary lipid metabolizers in the CNS. They also express proteins related to lipid sensing, such as transporter CD36 and peroxisome proliferator-activated receptor gamma, an important lipid-activated nuclear receptor that regulates transcription of numerous genes, including some involved in lipid metabolism (140, 141). In addition, astrocytic production of ketone bodies from fatty acids and their release to neurons could be one way by which an excess of fatty acids is signaled to metabolic neuronal circuits. Recent evidence suggests an increase in fatty acid β-oxidation in hypothalamic astrocytes from obese mice fed a HFD, as well as a role for tanycytes in restricting the passage of saturated fatty acids into the hypothalamus (142).

Although the brain produces lipids, it also has mechanisms to transport them from the bloodstream, but how they go through the BBB is not yet fully understood. Short and medium chain fatty acids appear to enter the CNS by simple diffusion through the plasma membrane (143). In contrast, long chain fatty acids (>12 carbons) need transporters to cross the BBB (144), with several fatty acid transport proteins (FATP) and fatty acid binding proteins (FABP) having been identified (145). *In vitro* studies indicate that FATP-1, FATP-4, and FABP-5 are the major isoforms expressed in microvessel endothelial cells and the gray matter of the human brain (145, 146). When the fatty acid translocase (FAT) CD36 is knocked-out in mice (CD36−/−), the uptake of monounsaturated fatty acids is significantly decreased, with no effect on polyunsaturated fatty acid uptake (147). In the CNS, CD36 is expressed in endothelial cells, microglial cells, astrocytes, and in ventromedial hypothalamic neurons (148–151). Although it is not the most highly expressed FATP, studies indicate that CD36 is responsible for fatty acid sensing in the hypothalamus and is thus important for the control of energy homeostasis (136, 137, 152).

In addition to the passage of free fatty acids through the BBB, lipids can also enter or exit the CNS as lipoproteins. This process is mediated by apolipoprotein E (ApoE) interacting with lipoprotein receptors (153). In the CNS, ApoE is expressed in astrocytes and tanycytes and its levels are upregulated by both leptin and TH (154, 155), with this process being involved in the regulation of food intake and energy balance (156).

### Hormone Transport and Signaling

#### Leptin

Leptin is an anorexigenic hormone that exerts part of its effects by inhibiting orexigenic neurons and activating anorexigenic neurons in the hypothalamus (157–159). It also has a role in the regulation of systemic lipid and glucose metabolism (160, 161). The leptin (or obesity) receptor (ObR), which has six isoforms, is highly expressed in brain endothelial cells, astrocytes and tanycytes (162–165), and endothelial and astroglial cells have been studied in attempt to unravel the mechanisms of leptin transport into the brain (163, 166). However, González-Carter and colleagues have recently reported that, in a human *in vitro* BBB model, leptin–ObR interaction is not necessary for the transport of this hormone across the BBB. They propose that lipoprotein receptor-related protein-2, expressed in endothelial cells at the BBB, is responsible for the passage of leptin across the BBB (167). Increasing evidence points to the BCSFB as the main pathway for entry of leptin into, at least, the hypothalamus (165, 168) (Figure 2).

The median eminence, a circumventricular organ close to the third ventricle, is the first site in the brain reached by blood-borne leptin (165). After an intraperitoneal leptin injection, there is a 1–2 h lag between the activation of leptin signaling pathways in the ventral and dorsal nuclei of the hypothalamus. This time-lag disappears when leptin is administered intracerebroventricularly, instead of intraperitoneally (169), suggesting that leptin transport from blood to the CSF is an important step in the action of this hormone in the brain and that it involves a delay in circulating changes reaching central target sites. Moreover, this process appears to be a finely regulated step in the control of energy balance as tanycytes act as “gatekeepers” for the passage of leptin into the mediobasal hypothalamus. Evidence suggests that leptin is first taken up by tanycyte processes in contact with the fenestrated capillaries at the median eminence (165) and that this uptake requires the activation of ObRb and the internalization of leptin by clathrin-coated vesicles (165). According to research carried out by Vincent Prevot and his team, this process involves signal transducer and activator of transcription (STAT)-3, protein kinase B (PKB)/Akt, and extracellular signal regulated kinase (ERK) phosphorylation, but is janus kinase-2 independent (165). Leptin is then transported toward the tanycyte cell body and, finally, released to the CSF and hypothalamic parenchyma (Figure 2) employing an ERK-dependent pathway (165). By using STAT-3 phosphorylation as an indicator of leptin signaling (170–172), Balland and collaborators reported that neutralization of leptin in the CSF impairs leptin signaling in mediobasal hypothalamic neurons (165), supporting the idea of the blood–CSF–hypothalamic gateway for leptin entry into the brain.

Taking into account the above mentioned studies, it appears that both endothelial cells and tanycytes contribute to the transport of leptin through the BBB and between different brain regions (163, 166, 167). In contrast, there is no clear evidence of the involvement of astrocytes in leptin transport, but a number of studies demonstrate that leptin signaling in astrocytes is important for energy homeostasis (173, 174).

Leptin transport into the brain is modulated by conditions including obesity and fasting, as well as metabolic factors. Obesity associated to HFD intake is reported to induce central leptin resistance. There are two main mechanisms or levels of leptin resistance suggested to occur: impairment of leptin transport into the brain (165) and reduction in the central response to leptin (175). Mice exposed long term to a HFD develop leptin resistance only when high levels of plasma leptin are reached (176). This suggests that hyperleptinemia is at least one of the causes of diet-induced leptin resistance. In addition, hypothalamic inflammation associated with diet-induced obesity could contribute to leptin resistance by altering the cellular networks and molecular pathways that control energy homeostasis (177). Nevertheless, recent studies suggest that leptin resistance does not imply a loss of responsiveness to endogenous leptin, but rather that there is a threshold above which exogenous leptin barely increases the response to leptin (178, 179). Glucose and insulin are reported to increase the transport of leptin across the BBB (180), while an increase in circulating triglycerides could impair leptin transport across the BBB (181). The latter suggests a possible mechanism for the reported reduction in leptin transport into the brain during fasting (182).

#### Ghrelin

Ghrelin is an orexigenic hormone produced and secreted in the stomach (183). It has similar targets as leptin in the CNS and also plays an important, but opposite, role in energy balance (184). There are two forms of ghrelin, acylated and unacylated, depending on the post-translational acylation with octanoic or decanoic acid (183, 185). This modification occurs mainly in the stomach, but there is evidence that it can also take place in target tissues (186). The acylated form of ghrelin exerts the majority of the metabolic effects of this hormone in the CNS and it binds more efficiently to the ghrelin receptor than the unacylated form (187). This receptor, also called the growth hormone secretagogue receptor 1a, is widely expressed in the hypothalamus (188). The mechanism underlying the passage of ghrelin across the BBB is not yet fully understood, but recent studies indicate that ghrelin possibly uses a similar route as leptin into the brain (189), i.e., through tanycytes in contact with the median eminence (Figure 2). Other studies indicate that this process is carried out by saturable transporters, at least for the acylated form, whereas transport of des-acyl ghrelin is not saturable (190). Entry of acylated ghrelin into the CNS is increased by serum triglycerides and fasting and is decreased in obese mice (191), in contrast with leptin transport. Diet-induced obesity is reported to impair the hypothalamic response to peripherally or centrally administered ghrelin (192). The role of unacylated ghrelin on metabolism is largely unknown, but an increasing number of studies reveal that des-acyl ghrelin has similar and opposite functions as the acylated form (193–196).

#### Insulin

Insulin is a pancreatic hormone directly involved in glucose metabolism and homeostasis. Within the brain, it acts to increase energy expenditure and reduce food intake and energy storage (197). Insulin binds to its receptor in the plasma membranes of endothelial cells at the BBB and is internalized following a saturable pathway (198, 199). Recent studies have shown that IRs in astrocytes are involved in the entry of this hormone into the CNS (120). Also, as mentioned above, insulin signaling in astrocytes is necessary for the regulation of systemic glucose levels (120). Insulin transport into the brain is enhanced by satiation hormones like cholecystokinin (200). Although estradiol is known to impair insulin’s actions in the brain, its effects appear to be unrelated to insulin transport (201). Some studies show that leptin increases insulin sensitivity in the hypothalamus at the molecular level (202), while others have found that leptin impairs insulin signaling in the brain (203). This discrepancy could be a matter of the experimental model employed, but further research is needed to understand the relationship between the effects of leptin and insulin at the level of the CNS. Leptin shares some signaling pathways with insulin, but the effects of these two hormones are not entirely parallel, as they exert opposite effects in some hypothalamic neurons (204). Saturated fatty acids induce insulin resistance in the hypothalamus (205), as has been previously described in peripheral tissues (206).

#### Sex Steroids

As hydrophobic molecules, estrogens, androgens, and progesterone can enter the brain by simple diffusion (207). Moreover, steroids are synthesized in the brain (208). These neurosteroids are produced in the CNS either from brain-borne cholesterol or from peripherally synthesized steroid precursors, like pregnenolone, deoxycorticosterone, and testosterone (209). The enzymes necessary for this synthesis are found in non-neuronal cells, including astrocytes, tanycytes, ependymal cells, and oligodendrocytes (210, 211), and in some neurons (212). As steroid hormones are known to regulate neurosteroid metabolism in glial cells (213–217) and also the expression levels of steroid receptors in the hypothalamus (218, 219), neurosteroids could have paracrine/autocrine functions within the brain.

Steroids and neurosteroids exert neuroprotective effects in the brain following brain injury, neurological disease, or inflammation (220–227). The expression of aromatase, the enzyme that catalyzes the conversion of testosterone into estradiol, is stimulated in reactive astrocytes after brain injury as a neuroprotective measure (228–230). Both microglial cells and astrocytes play an important role in the neuroprotective functions of steroids (231), as sex steroids diminish microglia reactivity (232–234) and astrocyte production of proinflammatory molecules (235–238).

Sex steroids, but specially estrogens, modulate energy homeostasis at the hypothalamic level decreasing food intake (239–241), increasing energy expenditure (242), and modulating the sensitivity to other metabolic hormones (243, 244). Their effect differs depending on the neuronal population (245, 246), but with an overall anorectic effect (247–249), although the underlying mechanisms are not yet fully understood. While nuclear estrogen receptors (ERs) are involved, especially ER α (247, 250–252), evidence indicates that estrogen responsive G-coupled membrane receptors can also regulate these effects (253, 254). The apparently contradictory results in the literature regarding the mechanism of action of estrogens on metabolism indicate a complex system for estrogens’ function in energy homeostasis, where the different ERs could be acting in combination (255). Moreover, the mechanisms of action used by estrogens in metabolic control could be sexually dimorphic (256). The involvement of neurosteroids in energy homeostasis remains unknown.

#### Thyroid Hormones

The role of TH in increasing the metabolic rate has been known for more than a century (257). The involvement of these hormones in the control of energy homeostasis at the central level is a more recent discovery (258, 259). They promote lipogenesis at the level of the hypothalamus, which eventually leads to brown adipose tissue thermogenesis (259) and blockage of TH signaling in the hypothalamus reverts this process, leading to weight gain without an increase in feeding (259). Clinical studies and animal models with a pathological excess of TH synthesis and secretion (hyperthyroidism) have shed light on TH action in the hypothalamus and control of energy balance (260). Most hyperthyroid patients have an increased appetite and food intake and decreased body weight (261). Moreover, these same symptoms that are observed in animal models of hyperthyroidism are associated with the upregulation of orexigenic neuropeptides AgRP and NPY and downregulation of anorexigenic neuropeptides derived from POMC in the arcuate nucleus (259). There is evidence that TH are involved in brain inflammation, promoting survival, and processes growth in microglial cells and also in astrocytes (262–264). TH are also involved in systemic glucose homeostasis and insulin sensing (265, 266).

The thyroid gland produces and secretes mainly tetraiodo-l-thyronine or thyroxine (T4), which is generally transformed into triiodo-l-thyronine (T3) through deiodination at the level of peripheral tissues (267). Thus, deiodinase enzyme expression in peripheral tissues is important for the control of TH actions (268), as they catalyze the transformation of T4 into T3 and of both hormones into reverse T3 (rT3) and 3,5-diiodo-l-thyronine (T2), respectively (269). These two last forms are usually considered inactive, although in the last few years new roles have been proposed for them and other non-classical TH (270).

Thyroid hormones enter the hypothalamus mainly through MCT-8 (271) and organic anion transporting polypeptide-1C1 (272) in rodents (273). These transporters are expressed in endothelial cells of the BBB and epithelial cells of the choroid plexus (274), besides neurons and glial cells of the hypothalamus (275–277). Tanycytes act as gatekeepers for TH at the BBB (61) (Figure 2). These cells express the enzyme deiodinase II (DII) (278–280), catalyzing the formation of hormone T3 from the prohormone T4. Tanycytes uptake T4 from the capillaries and release T3 to the extracellular space in the hypothalamus, where this hormone can exert its central actions (258, 259, 281) (Figure 2). Modulation of deiodinase expression is a key point in TH homeostasis. For example, DII expression in tanycytes is promoted in fasting conditions (282). DII-expressing tanycytes are in direct contact with AgRP/NPY-expressing neurons of the arcuate nucleus. Upregulation of DII results in an increased production of T3, which activates AgRP/NPY neurons and, therefore, feeding behavior (283, 284). Tanycytes also express deiodinase III (DIII), which deiodinates T4 into reverse T3 which is biologically inactive, and T3 into T2. TH is important in the adaptation to different photoperiods in seasonal animals were, for example, there is a decrease in food intake and body weight during short photoperiods. The study of hypothalamic metabolism of TH during photoperiodic changes in seasonal mammals has shown that the there is an upregulation of DII during periods of long days, which would increase the levels of T3. In Siberian hamsters an upregulation of DIII in tanycytes has been shown to occur during short photoperiods, lowering active T3 levels (285). The retinoic acid pathway in tanycytes appears to be similarly regulated by photoperiodicity and also leads to modifications in energy expenditure (286, 287).

Thyroid hormone signaling usually occurs through nuclear thyroid receptors α and β (288) that function as transcription factors modulating gene expression (289). TH can also exert rapid non-genomic actions through membrane-associated receptors (290, 291). This signaling pathway could mediate TH effects on vasodilatation (292) and has been shown to be involved in neuronal excitability in the hippocampus (293, 294).

Centrally, THs control their own homeostasis in various ways, with non-neuronal cells having an important role, i.e., regulation of deiodinase expression (278) and inactivation of thyroid releasing hormone (295). Other hormones involved in metabolic control can enhance the secretion, synthesis, or sensing of TH, including leptin (296–298) and sex steroids (299–301).

### Metabolism of Nutrients

#### Glucose

Perivascular astrocytes take-up blood-borne glucose that then undergoes glycolysis or glycogenesis (112). Lactate produced from glucose or glycogen metabolism in these cells is released to the extracellular space and enters neurons to be used as energy, constituting their primary energy source as suggested by some studies (302, 303). However, the question about the identity of the main energy source for neurons—lactate or glucose—is still debated. Tanycytes can metabolize and sense glucose in a similar manner (109).

Glucose storage as glycogen in astrocytes provides a way to guarantee energy release to neurons when it is needed, i.e., when faced with a raise in neuronal activity (304), by production of lactate from glycogenolysis. Several factors can regulate glycogen production and utilization in astrocytes, with insulin, insulin-like growth factor (IGF)-1 (305, 306), and leptin (203, 307) increasing their production of glycogen. More recently, ghrelin has been reported to possibly promote glycogenolysis in hypothalamic neurons (122).

#### Lipids and Ketone Bodies

It has been suggested that some fatty acids, like erucic acid (308, 309), suffer metabolic changes as they cross the BBB, whereas others do not (310, 311). Studies indicate that lipoproteins are hydrolyzed as they cross the BBB by the enzyme lipoprotein lipase associated to the membrane of endothelial cells (312–316).

In the absence of glucose and when glycogen stores are exhausted, such as in fasting conditions, astrocytes increase their uptake and utilization of fatty acids (136, 317, 318), which enter the mitochondria through carnitine palmitoyltransferase-1 to undergo β-oxidation (319). In the mitochondria, the enzymes 3-hydroxy-3-methylglutaryl-CoA synthase and lyase (320–322) transform fatty acids into β-hydroxybutyrate, a ketone body (323). Ketone bodies produced from this metabolic pathway are used by astrocytes themselves for fuel or secreted to be used by neurons and other glial cells (318).

### Neurogenesis

Glial cells were first reported to participate in neurogenesis during development (324–326), but it later became apparent that they are also involved in this process in adulthood (327). In the developing hypothalamus of the rat, the birth of metabolically important neurons occurs between embryonic days 10.5 and 18.5 (328–330). Environmental changes during this period, including nutritional and hormonal disturbances, can modulate the normal process of hypothalamic neurogenesis and have an impact on later neuroendocrine function (330–332). For example, HFD intake by pregnant dams stimulates the proliferation, differentiation, and migration of orexigenic neuronal precursors and increases the density of orexigenic neurons at the level of the paraventricular nucleus in the offspring. This increase in the number and density of appetite-stimulating neurons and orexigenic neuropeptide expression leads to increased appetite, body weight, and propensity to develop obesity later in life (331).

The clear demonstration, as well as its acceptance by the scientific community, of neurogenesis in the adult hypothalamus is relatively recent and there is still much to be learned. Tanycytes form part of the pool of neuroprogenitor cells in the hypothalamus and these precursors are capable of differentiating into not only neurons, but also astrocytes both during development and in the adult brain (333, 334). These specialized glial cells form an important neurogenic niche in the vicinity of the median eminence (333) and can proliferate and differentiate under basal conditions and when stimulated by growth factors such as IGF-1 (57, 95), fibroblast growth factor (FGF)-2 (335), FGF-10 (336, 337), or even vitamins, as tanycytes have been shown to express receptors, transporters, and other components of the vitamin A and C pathways (286, 287, 334, 338). FGF-10 positive tanycytes are reported to be important neural progenitors for NPY neurons in the arcuate nucleus, a function that may continue even during adulthood (337, 339). In addition, other isoforms of FGF are known to play a role in glucose homeostasis, inhibition of food intake, and body weight (340–343), with a possible involvement of glial cells (344–347). Although the generation of newborn neurons in the postnatal hypothalamus takes place at lower rates than during the embryonic period, it is physiologically relevant and has been shown to be regulated by diverse factors, including hormones and growth factors such as estradiol (332), FGF (335), and IGF-1 (348). Moreover, the nutritional status and dietary intake of an individual can modulate neurogenesis in hypothalamic metabolic circuits even in the adult animal (329, 333, 349–351).

The neurons composing the hypothalamic metabolic circuits experience a turnover rate such that approximately half of these cells are reported to be replaced between 4 and 12 weeks of age in mice (329). Diet-induced obesity suppresses this remodeling, at least in part, due to a decrease in actively proliferating cells in the hypothalamus with caloric restriction reversing this effect (329). Voluntary exercise is also reported to induce hypothalamic neurogenesis (352, 353). The effect of nutrient intake on the adult hypothalamus may be anatomically specific, as diets rich in fat are reported to inhibit neurogenesis in the mediobasal hypothalamic parenchyma (333), but to promote it in the median eminence in female mice (333). The enhanced neurogenesis that occurs at the level of the median eminence is suggested to be involved in the restoration of neurons that are damaged as a consequence of HFD intake (333); hence, impedance of this process could amplify the derogatory effects of poor dietary habits. There is tantalizing data indicating that hypothalamic neurogenesis in response to HFD differs between males and females (332, 333, 354), but whether this is involved in the sexually dimorphic metabolic response to HFD and weight gain requires further investigation.

Astrocytes are involved in the regulation of neuronal differentiation, proliferation, and synaptogenesis during development (3, 355). Microglia also actively participate in neurogenesis, both during development and adulthood, with most studies being performed in the hippocampus (356). Microglia not only phagocytize cells undergoing apoptosis in proliferative zones, but they also produce factors that can either inhibit or stimulate neuroprogenitor cells. The cross-talk between microglia and neuroprogenitor cells is an active area of investigation as this is a finely tuned process where these cells continuously interchange information (356). However, less is known regarding the role of astrocytes and microglia in neurogenesis in metabolic circuits of the adult animal.

Diet not only affects neurogenesis in the hypothalamus, but also in other brain areas such as the hippocampus, an area known to maintain active neurogenesis even in the adult (357). In the dentate gyrus of the hippocampus, HFD intake impairs neurogenesis (358) in addition to producing oxidative stress and lipid peroxidation (357). Palmitic acid (PA), a saturated fatty acid that is a major component of the majority of HFDs, was shown to reduce the proliferation of the neuroprogenitor cells (359) and the levels of brain-derived neurotrophic factor, indicating that PA-rich diets impair neurogenesis in the hippocampus. Caloric restriction and exercise increase neurogenesis in the hippocampus (350, 360, 361) and this has been associated with the anti-depressive effects of exercise (360).

### Synaptogenesis, Synaptic Plasticity, and Synaptic Transmission

Astrocytes, in addition to participating in neuronal proliferation and differentiation, also regulate synaptogenesis during development (3, 355). In the hypothalamus, the neonatal and early prenatal hormonal and nutritional environments can affect the synaptic connectivity of metabolic circuits (189, 362). Astroglial coverage of neuronal cell surfaces has been shown to be inversely correlated with the number of synaptic inputs to their somas, with this astroglial ensheathment/synaptic input arrangement being physiologically relevant in the neuroendocrine hypothalamus (363–367). Thus, changes in astrocyte numbers or morphology in the hypothalamus might be expected to modify synaptic inputs both during development and in later life.

The generation and maturation of astrocytes is not fully complete until the third postnatal week in rodents (368, 369), so variations in the physiological levels of specific metabolic hormones or signals during early life could affect the development of these cells. For example, neonatal overnutrition and modifications in leptin levels or signaling affect the number and morphology of astrocytes in the arcuate nucleus in adulthood (121, 173, 370). The leptin peak that takes place between postnatal days 5 and 13 in rodents is essential not only for neuronal outgrowth and maturation, but also astrogenesis (368, 371, 372) and astrocyte development (373, 374). The timing and magnitude of this leptin surge can be modified by nutrition (371, 375, 376), as well as other conditions such as stress (377) and is one mechanism by which these early environmental influences can have long-term effects on metabolism.

Maternal dietary intake and body weight during gestation and lactation can also influence metabolic circuit formation in the offspring, including the astroglial ensheathment/synaptic input arrangement. For example, newborns from mothers fed a HFD during gestation and lactation have increased astroglial ensheathment of POMC neurons that is associated with a decrease in the resting mini inhibitory post synaptic currents of these neurons (121). The response of these POMC neurons to changes in glucose concentrations was also shown to be modified (121). Hence, alterations in the early nutritional environment could imply the modification of the appropriate development of neuron–glial interaction of metabolic circuits and therefore affect long-term metabolism.

Microglia are involved in synaptogenesis throughout the brain (378, 379); however, there is little information regarding the specific effects of microglia on the development of the synaptic interactions of metabolic circuits. These glial cells have been shown to have an active role in the sexual differentiation of behavior and masculinization of the brain (380), suggesting that they may indeed be important for the development of endocrine circuits and possibly the sexual differentiation of some of these systems.

Modifications in the synaptic connectivity of metabolic circuits occur in postnatal life in response to metabolic and hormonal signals (241, 381–384) and are most likely involved in the adaptation to changes in energy inputs/conditions in attempt to maintain metabolic homeostasis, with astroglia participating in these synaptic rearrangements. HFD induced-obesity is associated with an increase in the glial coverage of both POMC and NPY cell bodies in the arcuate nucleus, which is coincident with a decrease in the number of synaptic inputs to the perikarya of these neurons (384). However, there is a decline in stimulatory inputs to NPY neurons and of inhibitory inputs to POMC neurons (384), which would result in an overall decline in orexigenic signaling. When first given a HFD, rodents experience a phase of hyperphagia that is normally followed by an attenuation of this rise in energy intake. The levels of polysialic acid (PSA) are rapidly increased in the arcuate nucleus in response to HFD (385). This cell-surface glycogen can attach to cell membrane proteins to weaken cell–cell interactions and facilitate synaptic reorganization (386). If PSA is enzymatically removed from neural-cell adhesion molecule (NCAM) in the hypothalamus, HFD induced modifications in metabolic circuits can be blocked and the adaptation to HFD-induced hyperphagia attenuated (385). In addition, studies in photoperiodic models have shown that PSA and NCAM levels in tanycytes are reduced during short photoperiods in conjunction with vimentin levels, modulating the plasticity for tanycyte connections with arcuate neurons (387).

Diverse hormonal/metabolic signals could be involved in these structural modifications, including leptin. This hormone rapidly induces synaptic changes in metabolic circuits (381), with some of these effects being mediated through astrocytes. These glial cells express different isoforms of ObR (163, 164), with the expression of this receptor being increased in astrocytes of obese rodents (163). Leptin can modify astrocyte morphology, inducing changes in the length and number of primary astrocytic projections and astroglial coverage of hypothalamic neurons (173, 388). The lack of leptin signaling due to the knock-out of this receptor in astrocytes changes synaptic inputs to POMC and NPY neurons, resulting in modifications in the function of these metabolic neurons and rendering the animals less susceptible to the effects of leptin (173). However, it remains unclear as to the mechanisms involved in the changes in neuronal/glial interactions, including identification of the initial step that triggers these morphological modifications.

Astrocytes modulate neuronal transmission by controlling glutamate concentrations in the synaptic cleft, which also plays an important role in preventing excitotoxicity (389). Leptin and ghrelin modulate glutamate uptake by these glial cells (121, 122) and could thus affect stimulatory signaling in metabolic circuits through this mechanism. Astrocytes also actively participate in synaptic transmission and plasticity by releasing gliotransmitters, including adenosine, ATP, d-serine, glutamate, and tumor necrosis factor α that directly activate postsynaptic receptors and by altering neurotransmitter release from presynaptic neuronal elements to induce short-term plasticity and to modulate synaptic efficacy (12, 390–393). Adenosine release by astrocytes inhibits the firing rate of AgRP neurons and food intake, modifying the response to metabolic hormones such as ghrelin (394).

### Inflammatory Response

The inflammatory response to infection, foreign substances, mechanical damage, or any situation that could damage neurons is one of the best studied functions of glial cells (231, 395–398). However, the description of hypothalamic inflammation in obesity, as well as its association with the development of secondary complications, is more recent. In 2005, the group of Licio Velloso reported that inflammatory pathways were activated in the hypothalamus in HFD-induced obese rats (399). This same group went on to demonstrate that this hypothalamic inflammation was involved in the disruption of systemic glucose homeostasis (35). Numerous studies have since reported the link between hypothalamic inflammation and obesity-related comorbidities (36, 400–404). Hypothalamic inflammation is reported to be associated with the development of insulin resistance and type 2 diabetes (405) and increased cell death in the hypothalamus (329). Most studies analyzing hypothalamic inflammation have employed HFD-induced obesity models and suggest that dietary factors are involved in at least part of the inflammatory response. Indeed, hypothalamic inflammation is reported to occur even before an increase in adiposity or systemic inflammation are detected (36) and central administration of saturated fatty acids directly activates inflammatory signaling mechanisms in the hypothalamus (35, 406). However, increased weight gain can occur in response to genetic, epigenetic, and excess energy intake that is not due to increased fat consumption and the hypothalamic inflammatory/gliosis response differs depending on the underlying cause of weight gain (121, 195, 407, 408). These differential responses are most likely the result of dietary signals and the changes in metabolic signals associated to weight gain acting on both microglia and astrocytes. Sex may also be a factor, as the hypothalamic inflammatory response to chronic HFD-intake is reported to differ between males and females, with males being more susceptible (409). This could result from the greater rise in PA levels in the CNS of male mice compared to females, even though there is no sex difference in circulating fatty acid levels (410).

Inhibition of hypothalamic inflammation is reported to blunt or block the development of obesity-associated complications (400, 403) and dietary restriction can reverse central inflammatory processes (411–415). Exercise also protects against HFD-induced hypothalamic inflammation (416).

#### Microglia in Hypothalamic Inflammation

Microglia, the innate immune cells of the CNS, are the first line of defense in response to foreign substances (417, 418) and are activated in response to saturated fat consumption (36, 403, 408, 419). Indeed, these glial cells are suggested to dictate the inflammation that occurs in response to saturated fats (419). Microglia can also be activated when weight gain is due to excess intake of a normal diet and due to high fat intake, (402), indicating that not only dietary signals are involved. Leptin stimulates the release of inflammatory cytokines from microglia (420), suggesting that hyperleptinemia could be involved in microglial activation in obese subjects.

#### Astrocytes in Hypothalamic Inflammation

Astrocytes also respond to HFD intake (36, 384, 421) and can be directly activated *in vitro* by fatty acids (408, 409, 422). Hyperleptinemia associated with weight gain may also participate in the activation of glia in situation of obesity (121, 173, 388, 408, 423). Indeed, ob/ob mice, which are dramatically obese due to the genetic lack of leptin, do not exhibit astrogliosis and leptin-induced weight loss actually increases astrocytic profiles in the hypothalamus of these animals (408). However, we have found that in some situations of increased weight gain, such as increased carbohydrate intake in the form of sucrose, astrocytic markers may actually be decreased (407).

The astrogliosis response to HFD differs between males and females, as does the *in vitro* response to PA (409). The protective effects of estrogens are exerted through ERα in astrocytes (424), with estrogens protecting against PA activation of astrocytes *in vitro* (409). Morselli et al demonstrated that HFD-intake reduces hypothalamic ERα levels in males, but not in females, which may be involved in the decreased protection against diet-induced obesity in males.

Astrocytes have also been implicated in determining the preference for a HFD, with this mechanism involving cannabinoid receptor 1 (CB1) (425, 426). The intake of a HFD induces the preference for this type of diet and this appears to involve the production of endocannabinoids in the hypothalamus (426). Leptin signaling in astrocytes is regulated by CB1, with disruption of CB1 in these glial cells resulting in the inability of leptin to regulate glycogen storage (307) and thus possibly affecting central energy storage and glucose sensing/signaling.

## Conclusion

**Figure 3:**
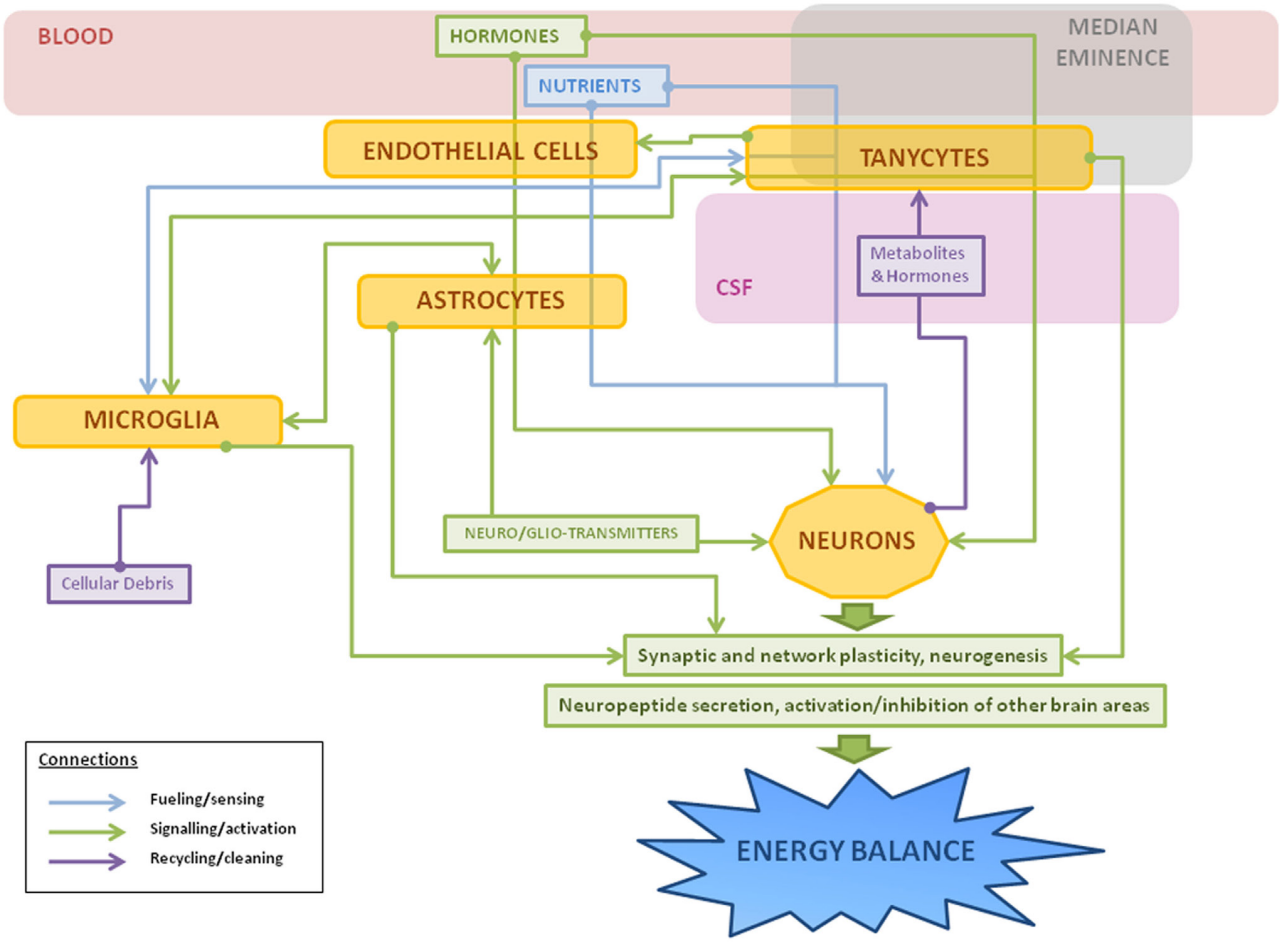
**Schematic representation of the main roles of hypothalamic non-neuronal cells in metabolism**. Thin arrows represent the different connections between cells: fueling and sensing of nutrients (in blue); signaling through gliotransmitters, neurotransmitters, or other factors (in green); and recycling of molecules or cleaning of cellular debris (in purple). Hormones and nutrients from the bloodstream pass through one or more types of non-neuronal cells before reaching neurons, sometimes suffering metabolic changes during the process. In response to a metabolic imbalance (excess of saturated fatty acids, hyperleptinemia, etc), microglial cells change to an activated state, releasing inflammatory factors, such as cytokines, and activating astrocytes as a neuroprotective measure. If the insult continues, it can lead to gliosis, hypothalamic inflammation, and neuronal damage.

It is clear that non-neuronal cells are fundamental for the correct functioning of metabolic circuits, beginning with the essential process of regulating the nutrients and signals that reach these neurons. These cells are not only involved with the development, maintenance, and protection of their neuronal neighbors, but participate in all aspects of neuronal function (summarized in Figure 3). In the hypothalamus, numerous studies have shown how non-neuronal cells play an active role in the control of metabolism and in the pathological outcomes of poor metabolic control. Although the advances in laboratory techniques and genetically engineered animal models have increased our knowledge in this field, there is yet much to be learned regarding the mechanisms involved. Studies directed at developing markers to further identify different populations or subclasses of glial cells are of great importance in order to better understand the vast roles that these cells play in the different physiological functions controlled by the CNS. This increased knowledge will also hopefully add to our understanding of pathophysiological processes and future targets for treatments, including that of obesity and its secondary complications.

## Author Contributions

All authors have contributed to the writing and editing of this review. Figures were designed and made by AF-R.

## Conflict of Interest Statement

The authors declare that the research was conducted in the absence of any commercial or financial relationships that could be construed as a potential conflict of interest.
